# Acute Hemolytic Anemia Due to Glucose-6-Phosphate Dehydrogenase (G6PD) Deficiency Triggered by Helicobacter pylori Quadruple Therapy in a Jehovah’s Witness: A Case Report

**DOI:** 10.7759/cureus.102977

**Published:** 2026-02-04

**Authors:** Amandeep S Dhami, Purvesh Koladiya, Fatin Sahhar

**Affiliations:** 1 Department of Internal Medicine, Wayne State University School of Medicine, Detroit, USA; 2 Department of Family Medicine, Wayne State University Detroit Medical Center, Detroit, USA

**Keywords:** anemia management, bismuth quadruple therapy, erythropoietin, g6pd deficiency, ganzoni equation, helicobacter pylori, hemolytic anemia, intravenous iron, jehovah’s witness, oxidative stress

## Abstract

Glucose-6-phosphate dehydrogenase (G6PD) deficiency, the most common erythrocyte enzymatic disorder, predisposes patients to oxidative stress-induced acute hemolysis. Affected patients are at risk of developing hemolysis in the setting of triggers such as certain drugs, infections, or foods (fava beans). Although some drugs like antimalarials (primaquine, tafenoquine), sulfonamides, and dapsone are well-known triggers of hemolysis, reports of this adverse effect are rare with standard bismuth-based quadruple therapy for *Helicobacter pylori*. In this report, we present a 65-year-old male who developed profound anemia (hemoglobin 5.8 g/dL, drop of 4.4 points from baseline) within hours of initiating metronidazole- and tetracycline-containing quadruple therapy. Laboratory evaluation revealed reduced G6PD enzyme activity (3.8 U/g; normal 9.9-16.6 U/g), confirming acute hemolytic anemia secondary to G6PD deficiency. Notably, the patient was a Jehovah’s Witness and declined all blood products, requiring atypical management. Quadruple therapy was discontinued, and the patient was managed with intravenous fluids, iron supplementation, and erythropoietin (EPO) therapy, resulting in gradual hematologic recovery. Regarding *H. pylori *treatment, the patient was initiated on triple therapy (clarithromycin, amoxicillin, proton pump inhibitor), without recurrence of hemolysis. This case highlights the rarity of G6PD deficiency as an etiology of unexplained hemolysis during initiation of *H. pylori* eradication therapy and underscores the importance of unique management strategies in the setting of blood transfusion refusal. Clinicians should maintain a high suspicion for G6PD deficiency in high-risk populations and exercise caution when prescribing oxidative regimens for *H. pylori* in these patients.

## Introduction

Glucose-6-phosphate dehydrogenase (G6PD) is the first and rate-limiting enzyme of the pentose phosphate pathway (PPP), a cytosolic pathway responsible for producing nicotinamide adenine dinucleotide phosphate (NADPH) and nucleic acid precursors. The oxidative phase begins with G6PD catalyzing the oxidation of glucose-6-phosphate to 6-phosphogluconate, while reducing NADP+ to NADPH [1]. NADPH is crucial to the antioxidant system, as it provides reducing power to glutathione reductase to generate reduced glutathione, which detoxifies reactive oxygen species (ROS) and protects erythrocytes, in which the PPP is the sole source of NADPH [2-3].

Globally, 400 million individuals are affected by G6PD deficiency, making this the most prevalent enzymopathy. The highest-risk patient populations are found in Sub-Saharan Africa, Mediterranean Europe, South-East Asia, and Latin America, all historically malaria-endemic areas [4]. Furthermore, within sub-Saharan Africa, ten malaria-endemic countries were found to have extreme variability and heterogeneity in the prevalence of G6PD deficiency, ranging from less than 1% in parts of Mali to as high as nearly 24% in Nigeria. Notably, males are more commonly affected, likely due to the X-linked inheritance of the G6PD gene [5]. Well-documented triggers of hemolysis in these patients are important to be aware of; however, it is imperative to also consider rare triggers, such as standard quadruple therapy for *Helicobacter pylori* infection.

*H. pylori* first-line treatment is standard bismuth-based quadruple therapy consisting of a proton pump inhibitor (PPI), bismuth, metronidazole, and tetracycline for 14 days, particularly when antibiotic resistance is unknown [6]. While typically well tolerated, it is important to recognize that both metronidazole and tetracycline have been linked to promoting oxidative stress. In preclinical models, high-dose metronidazole therapy administered to albino mice led to elevated cardiac injury markers secondary to oxidative stress with histopathological evidence of myocyte degeneration, fibrosis, and necrosis. Furthermore, a significant increase in DNA fragmentation was observed in metronidazole-treated mice versus the control [7]. Human evidence for these effects is limited. We present a case of hemolysis triggered by *H.* *pylori* quadruple therapy in a patient with previously undiagnosed G6PD deficiency and management complicated by blood transfusion refusal due to the patient’s religious beliefs. This case underscores a rare trigger of hemolysis and distinctive management considerations when blood transfusion is refused in acute anemia.

## Case presentation

A 65-year-old male with a past medical history of chronic kidney disease stage IIIb and hepatic steatosis presented to the emergency department accompanied by his wife with complaints of nausea, vomiting, poor oral intake, and epigastric abdominal discomfort, all present for about two weeks but worse over the two days before arrival. Furthermore, the patient’s wife noted that he appeared confused over the past 24 hours and lethargic, stating that he had not gotten out of bed for most of the day. He reported a fall while getting out of bed the day before arrival, during which he hit his head, but denied any prodromal symptoms, stating that he simply felt off balance. There was no other pertinent medical or surgical history. Regarding social history, the patient endorsed occasional alcohol use but none recently due to feeling ill. He denied any history of heavy alcohol use and denied tobacco or recreational drug use. The patient is employed as an electrical engineer and lives with his spouse.

Per the patient’s spouse and review of external documentation in the electronic medical record (EMR), the patient was admitted to a different hospital system for similar complaints two weeks prior but did not exhibit lethargy or confusion at that time. The patient stated that he was discharged from the hospital on Protonix and folic acid supplementation and was instructed to follow up in the gastroenterology (GI) clinic for *liver problems*. Per chart review, the patient underwent esophagogastroduodenoscopy (EGD) during that admission, which demonstrated moderate erythematous mucosa with erosion in the gastric body and erythematous mucosa in the duodenum; pathology was positive for *H. pylori*. The patient and his spouse were unaware of the positive biopsy results and reported that the only medications he was taking were the two prescribed at discharge. He also underwent a contrast-enhanced CT scan of the abdomen and pelvis during that admission, which revealed hepatic steatosis, colonic diverticulosis without diverticulitis, an enlarged prostate, a small hiatal hernia, and a left renal cyst. Since discharge from the other hospital approximately two weeks prior, the patient’s symptoms gradually worsened, with multiple episodes of non-bloody emesis, fatigue, lethargy, and confusion, prompting his wife to bring him back to the hospital. 

On initial examination, the patient appeared slightly lethargic and slow to respond but was alert and oriented ×3. He was hemodynamically stable, with initial vital signs showing a blood pressure of 115/75 mmHg, a heart rate in the 90s beats per minute, afebrile at 36.7 °C, a respiratory rate of 18 breaths per minute, and an oxygen saturation of 97% on room air. The cardiopulmonary examination was unremarkable. Abdominal examination revealed a protuberant, soft, non-tender, mildly distended abdomen without guarding or rigidity. A chronic umbilical hernia was noted. Neurological examination revealed no focal deficits, with cranial nerves II-XII intact. Mild bilateral scleral icterus was noted, without jaundice.

The initial workup included laboratory analysis and imaging, including a CT scan of the head due to the recent fall with head injury and an abdominal ultrasound. The CT head was negative for any acute findings. The abdominal ultrasound demonstrated heterogeneous, dense echotexture and mildly increased echogenicity of the liver parenchyma, without gross morphologic features of cirrhosis. The gallbladder was well visualized and physiologically distended with bile. No intraluminal stones, sludge, or masses were identified. Markedly limited Magnetic resonance cholangiopancreatography (MRCP) was markedly limited by artifact, resulting in suboptimal evaluation of the liver and pancreas; a non-contrast CT scan of the abdomen showed no definite pancreatic mass but remained limited without IV contrast, with mild inflammatory changes adjacent to the sigmoid colon suspicious for early diverticulitis, a distended gallbladder without stones, and a likely benign 3 cm right renal cyst. Remarkable initial laboratory findings included a baseline hemoglobin of 10.1 g/dL with a hematocrit of 31.2%, a creatinine of 2.14 mg/dL (elevated from a baseline of 1.8 mg/dL), and a total bilirubin of 3.54 mg/dL with a direct bilirubin of 1.74 mg/dL, consistent with mixed hyperbilirubinemia. Liver function tests were elevated, with alanine aminotransferase (ALT) of 190 U/L and aspartate aminotransferase (AST) of 66 U/L, while alkaline phosphatase was normal. Low-density lipoprotein (LDL) cholesterol was elevated at 142 mg/dL, and triglycerides were elevated at 173 mg/dL. Iron studies revealed significantly low iron levels of <10 ug/dL (normal 50-212), hyperferritinemia, and an elevated unsaturated iron-binding capacity (UIBC) of 1,350 ug/dL (normal 155-355). Urinalysis was remarkable for dark-appearing urine, urobilinogen of 3.0 (normal 0-1), and negative leukocyte esterase, nitrites, and blood.

The patient’s care plan consisted of starting 14 days of bismuth-based quadruple therapy for *H. pylori*, providing symptomatic relief, and having GI follow the patient’s case for further recommendations. Two days after the initial admission, the patient received his first evening doses of metronidazole 500 mg and tetracycline 500 mg together at 21:12, after having received bismuth and a proton pump inhibitor earlier that day. On the morning of hospital day three, the patient received his second dose of tetracycline at 02:30 and metronidazole at 05:40. Later that same day, the patient received a third dose of tetracycline at 09:37 and metronidazole at 13:44. On the morning of hospital day three, laboratory studies drawn at 05:21 revealed a significant drop in hemoglobin from 10.2 to 5.8, occurring approximately eight hours after the initial doses of metronidazole and tetracycline. The timeline of pertinent laboratory results is given in Table 1. Quadruple therapy was immediately held, and he received no further doses. Two additional CBCs were drawn on hospital day three at 10:27 and 15:30, with hemoglobin values of 6.0 and 5.8, respectively, confirming acute anemia. During this episode, the patient remained clinically stable with an unchanged physical examination, was hemodynamically stable, and denied any bleeding, bruising, chest pain, shortness of breath, palpitations, dizziness, lightheadedness, or changes in fatigue or lethargy. 

**Table 1:** Timeline of laboratory trends. Pertinent laboratory results throughout hospital admission demonstrated acute hemolysis on day 3 of admission after the initial dose of metronidazole and tetracycline, with subsequent gradual hematological recovery following supportive care. MCV, mean corpuscular volume (femtoliters); WBC, white blood cell count (K/µL); LDH, lactate dehydrogenase (U/L); Hb, hemoglobin

Parameter	Reference range	Admission (Day 0)	Day 1	Day 2	Day 3	Day 4	Day 5	Day 9	Day 18	Day 23
Hemoglobin (g/dL)	13.3-17.1	10.1	10.2	-	5.8	5.5	5.4	7.3	8.8	9.8
Hematocrit (%)	38.9-49.7	31.2	31.1	-	18.5	16.9	16.9	23.8	27.7	30.7
MCV (fL)	81-98	94.8	96.1	-	97.4	98.3	97.7	105.1	-	98.4
Platelets (K/µL)	150-450	114	100	-	93	94	97	204	-	185
WBC (K/µL)	3.5-10.6	11.3	8.1	-	5.7	6.0	6.4	8.6	-	7.0
Total bilirubin (mg/dL)	<1.50	3.54	3.13	-	1.76	1.40	1.36	-	-	-
Direct bilirubin (mg/dL)	0.03-0.18	1.74	-	-	0.75	-	-	-	-	-
LDH (U/L)	140-271	-	186	-	-	179	-	-	-	-
Haptoglobin (mg/dL)	44-215	-	-	-	-	-	119	-	-	-
Reticulocyte count (%)	0.5-2.0	2.6	-	-	2.6	-	-	-	-	-
G6PD activity (U/g Hb)	9.9-16.6	-	-	-	3.8	-	-	-	-	-

The patient was offered a blood transfusion due to a rapid and significant decline in hemoglobin to below 7 g/dL; however, he declined, stating that he is a Jehovah’s Witness and does not accept blood products. He did agree to supportive therapies. In accordance with hematology recommendations, the patient was treated with intravenous epoetin alfa (EPO) 10,000 units daily from hospital day 4 through day 6, followed by three doses of EPO 20,000 units administered on hospital days 6, 8, and 9. Additionally, he received intravenous sodium ferric gluconate 250 mg daily for six days, from hospital day 3 through day 8, and high-dose folic acid 5 mg daily for five days, starting on day 3 of admission. The patient was also given intravenous fluids (normal saline) to support hydration and renal function. Blood draws were minimized, pediatric tubes were used when possible, and anticoagulation for DVT prophylaxis was held. The patient’s G6PD level was low at 3.8 U/g (normal range 9.9-16.6). Peripheral blood smear showed significant polychromasia, but no ghost cells or bite cells were observed. Direct antiglobulin testing, performed per hematology recommendations, was negative. On hospital day 4, the patient was started on *H. pylori* triple therapy with clarithromycin 500 mg BID, amoxicillin 1 g TID, and intravenous pantoprazole 40 mg BID, concurrent with blood count-supportive therapies. No further episodes of hemoglobin decline were noted.

**Figure 1:**
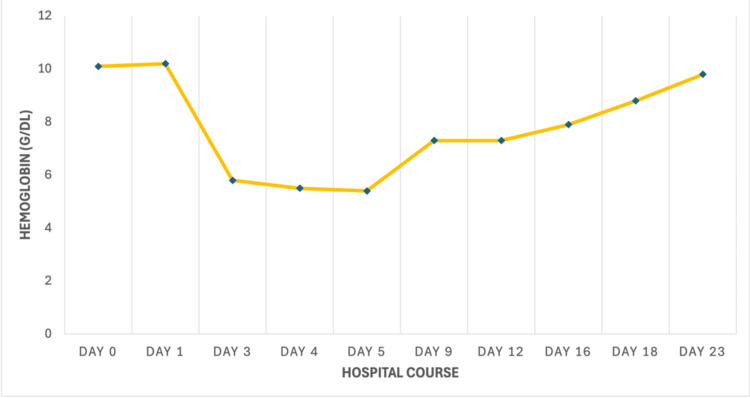
Hemoglobin trend during admission. Hemoglobin levels (g/dL) plotted by hospital day demonstrate an acute drop from baseline on day 3, persisting through day 5, followed by gradual recovery to baseline by the time of discharge with supportive care measures.

Over the course of the following week, the patient showed clinical improvement. He reported feeling more energetic than at admission, and his oral intake increased, as he was able to finish multiple meals each day. His wife also noted an improvement in his previously observed confusion. The patient tolerated *H. pylori* triple therapy well, with no recurrent episodes of hemolysis. After 26 days of hospitalization, the patient was discharged home with instructions for close follow-up with both gastroenterology and hematology. At discharge, his hemoglobin had nearly returned to baseline at 9.8 g/dL. The patient and his spouse received education regarding G6PD deficiency, including triggers to avoid, such as specific foods and medications (Figure 1).

## Discussion

This case describes a rare trigger of hemolysis in a patient with previously undiagnosed G6PD deficiency, with atypical management due to refusal of blood transfusion. The Ganzoni equation, originally described in 1970, calculates total body iron deficit to guide iron replacement dosing: Iron deficit (mg) = body weight (kg) × (target hemoglobin − actual hemoglobin) (g/L) × 2.4 + iron stores (mg) [8]. Using this equation, the patient’s total iron deficit was calculated at 2,154 mg. He received six days of 250 mg IV iron, plus an additional 125 mg dose, for a total of 1,625 mg during hospitalization, with further doses planned as an outpatient. Although the Ganzoni formula remains historically important and in use, more recent evidence, such as the FERGIcor study, has demonstrated that a fixed-dose IV iron regimen can be more effective in certain clinical contexts [9].

The Food and Drug Administration (FDA) recommends screening for G6PD deficiency in high-risk populations, such as individuals of African or Mediterranean ancestry, before initiating rasburicase therapy, which is a known oxidative trigger [10]. Similarly, G6PD testing is required prior to prescribing the antimalarials primaquine or tafenoquine [11]. Considering these mandatory guidelines, the question arises whether patients from ancestrally high-risk populations for G6PD deficiency should also undergo screening before initiating bismuth-based quadruple therapy for *H. pylori* eradication.

Our report highlights a rare and novel trigger of hemolysis in G6PD deficiency, although causality cannot be established from a single case. Retrospective chart reviews in hospital systems serving populations with a high prevalence of G6PD deficiency could help identify potential associations between hemolysis and the administration of metronidazole or tetracyclineAdditionally, prospective studies can be performed to assess the risk of hemolysis when prescribing these medications. These studies have the potential to alter management by either supporting preemptive G6PD screening or guiding the use of alternative therapies to prevent acute hemolytic anemia.

## Conclusions

Our report highlights bismuth-based quadruple therapy for H. pylori eradication as a rare trigger of hemolytic anemia in G6PD deficiency, successfully managed without blood transfusion in a Jehovah’s Witness patient. A high index of suspicion should be maintained when prescribing oxidative regimens in high-risk populations, even with less commonly documented triggers. This report highlights the unique management of acute hemolytic anemia with supportive therapies, including Ganzoni formula-guided intravenous iron repletion, erythropoietin, and fluids. Future investigations may alter management by establishing how significant a risk quadruple therapy holds in inducing hemolysis in G6PD-deficient individuals.

## References

[1] Ho HY, Cheng ML, Chiu DT (2007). Glucose-6-phosphate dehydrogenase--from oxidative stress to cellular functions and degenerative diseases. Redox Rep.

[2] Stanton RC (2012). Glucose-6-phosphate dehydrogenase, NADPH, and cell survival. IUBMB Life.

[3] Stincone A, Prigione A, Cramer T (2015). The return of metabolism: biochemistry and physiology of the pentose phosphate pathway. Biol Rev Camb Philos Soc.

[4] Nkhoma ET, Poole C, Vannappagari V, Hall SA, Beutler E (2009). The global prevalence of glucose-6-phosphate dehydrogenase deficiency: a systematic review and meta-analysis. Blood Cells Mol Dis.

[5] Taleb M, Brahim AT, Yebouk C, Khairy T, Ishagh B, Lyoussi B, Ghaber SM (2025). Epidemiology of glucose-6-phosphate dehydrogenase deficiency in 10 malaria-endemic countries in sub-Saharan Africa: a systematic review. Pan Afr Med J.

[6] Chey WD, Howden CW, Moss SF, Morgan DR, Greer KB, Grover S, Shah SC (2024). ACG clinical guideline: treatment of Helicobacter pylori infection. Am J Gastroenterol.

[7] Coşkun F, Yalçın E, Çavuşoğlu K (2024). Metronidazole promotes oxidative stress and DNA fragmentation-mediated myocardial injury in albino mice. Chemosphere.

[8] Ganzoni AM (1970). Intravenous iron-dextran: therapeutic and experimental possibilities. Schweiz Med Wochenschr.

[9] Evstatiev R, Marteau P, Iqbal T (2011). FERGIcor, a randomized controlled trial on ferric carboxymaltose for iron deficiency anemia in inflammatory bowel disease. Gastroenterology.

[10] (2025). U.S. Food and Drug Administration. Highlights of prescribing information: ELITEK (rasburicase). https://www.accessdata.fda.gov/drugsatfda_docs/label/2009/103946s5083lbl.pdf.

[11] Haston JC, Hwang J, Tan KR (2019). Guidance for using tafenoquine for prevention and antirelapse therapy for malaria - United States, 2019. MMWR Morb Mortal Wkly Rep.

